# An Asymptomatic Foreign Body in the Nose in an Eighteen-Year-Old Patient: Button Battery

**DOI:** 10.1155/2015/129851

**Published:** 2015-11-19

**Authors:** Merih Onal, Gultekin Ovet, Necat Alatas

**Affiliations:** Konya Education and Training Hospital, Department of Otorhinolaryngology, 42090 Konya, Turkey

## Abstract

Foreign bodies lodged in the upper airway are a common occurrence in children. Many unusual foreign bodies in the nose have been reported as foreign bodies like nuts, plastic toy parts, beads, and so forth. Most of these produce minimal morbidity but button batteries due to their early chemical disintegration require early surgical intervention. Here, we report a case of button battery lodged in the nose for several years with a symptom of nasal obstruction and chronic sinusitis.

## 1. Introduction 

Nasal foreign bodies are generally referred to otorhinolaryngologists [1]. Foul-smelling, purulent nasal flow and epistaxis are typically present in nasal foreign bodies. In particular, stuck nasal foreign bodies may cause morbidities such as chronic sinusitis, septal perforation, or necrosis of bones in the long term obstruction. The button battery is generally used in small devices such as hearing aids, watches, and electronic gadget. Their smooth and shiny surface possibly makes them interesting for children. A button battery in the nose is capable of tissue damage due to its electrochemical content. But sometimes it does not cause any ill effect [2] depending on several factors such as remaining voltage in the battery and chemical composition of the battery [3]. We report a case of 18-year-old young lady who was referred to our clinic with unknown nasal foreign body.

## 2. Case 

**Figure 1:**
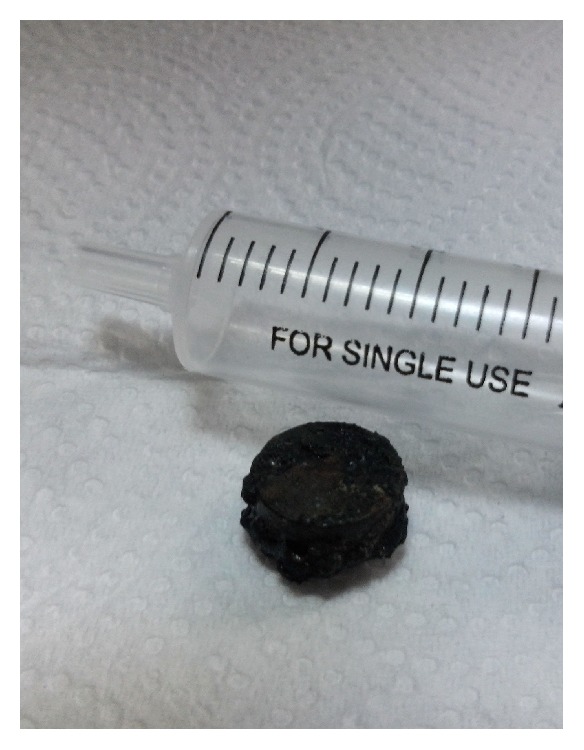
The button battery removed from the nose.

**Figure 2:**
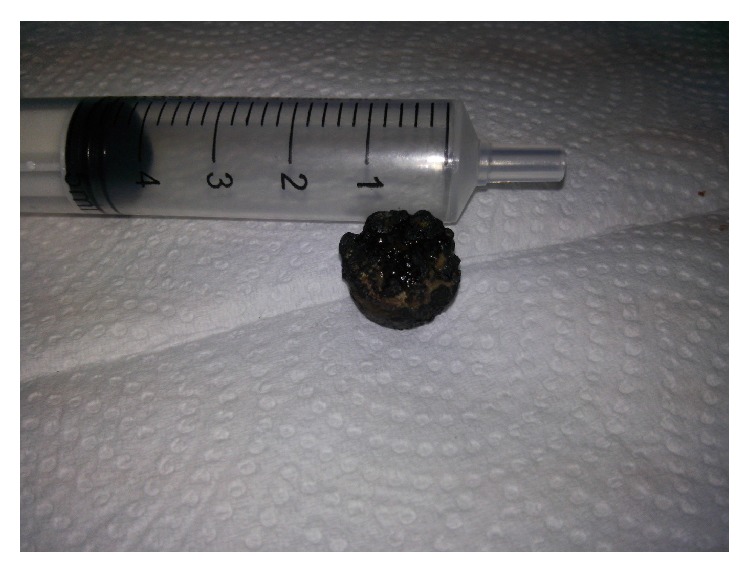
The button battery with its irregular darkish surface.

18-year-old female patient was referred to our clinic with a diagnosis of foreign body in the nose from another center. The patient had nasal congestion and rhinorrhea. She was admitted to doctor with these complaints many times before and acute sinusitis treatment was given. An otorhinolaryngologist noticed a foreign body in the right nasal passage on her recent admission. He tried to take it out but he failed. He referred the patient to us immediately. She had nasal septal deviation and a prominent maxillary crest on the right side. Between the crest and the hypertrophic inferior turbinate, a black-colored, rigid, and irregular foreign body was impacted. It was thought to be a rhinolith. Forward traction with a curved curette was attempted but the foreign body did not move. Then the foreign body was pushed from the bottom and pulled outward. The object had an irregular and darkish surface, but silver colored button battery can be distinguished in this structure (Figures 1 and 2). She could not remember when she put this button battery into her nose. There was maceration and hemorrhage but no necrosis or perforation was seen, where the battery was removed. Gel foam was placed. One week later, nasal endoscopy revealed a healed nasal mucosa covering the turbinate and septum with a patent nasal passage.

## 3. Discussion 

Cases of foreign body in the nose usually occur in childhood. Foul-smelling discharge and nasal bleeding are symptoms of nasal foreign body. Whatever the object is, it has to be removed as soon as possible when it is noticed [4]. In this case, unlike many other cases, the nasal foreign body may remain asymptomatic for a long time. Our patient had only a complaint of nasal stuffiness and sometimes a runny nose. She has been treated several times for sinusitis. Probably for the reason of posterior localization and the black color, the foreign body was undetected in previous anterior rhinoscopic examinations. In addition, no imaging method was performed also.

The first report of nasal button battery was published in 1986 [5]. The button battery may irritate the nasal mucosa and cause rhinorrhea. Generally, they are in alkaline structure leading to further chemical disintegration upon contact with saline nasal mucosal discharge. Thus, more capability of additional damage occurs in the nasal mucosa. Secondary to chemical, electrical, and mechanical trauma, ulceration and necrosis of the nasal mucosa and cartilage may be seen, and even perforation may occur. McRae and his colleagues' in vitro studies have showed that spontaneous leakage of electrolyte solution occurs in alkaline batteries which are exposed to moisture. This leaked structure penetrates tissues and causes liquefying necrosis [1, 6]. The current between the battery poles which causes tissue fluids hydrolysis and gives rise to corrosive hydroxides is the primary cause of tissue destruction [7]. The damage of the button battery depends on exposure time. Tong et al. [8] reported that damage to the nasal mucosa has been reported after as few as 3 h and damage leading to perforation after 7 h. Necrosis of the inferior turbinates has occurred also at 24 hours [8]. Foreign bodies in the nose often do not cause significant morbidity or mortality, but this is not valid for button battery cases. The immediate removal is necessary when noticed. Moreover, when a button battery is detected in the nose, saline or vasoconstrictors should not be used because they enhance mucosal necrosis by chemical interaction with alkaline battery [6, 9]. In our patient, button battery does not cause any damage to the nose interestingly. She did not remember when she put it into her nose. Probably the remaining voltage of the battery was diminished. Removal is best accomplished with a right angle hook or ear curette placed behind the battery to pull it forward and out as in our case [10].

## 4. Conclusion 

The button battery should be treated as a life threatening foreign body due to its electrochemical content and rapid tissue damage. Early suspicion and detection of accidental foreign body impaction are the key in the management of foreign bodies. The most effective management of foreign body is prevention. Because of rapid progression of tissue damage, early removal of button batteries must be required. Nonetheless, we see that a button battery can stay in the nose for long periods without specific complaints. But it should be removed when detected [4, 11].
